# Effects of *Beauveria bassiana* Alone and Combined with Yeast-Fermented Prebiotics on *Hypothenemus hampei*

**DOI:** 10.1007/s13744-026-01445-z

**Published:** 2026-10-28

**Authors:** Ana Paula Ananias Antunes, Bruno Henrique Sardinha de Souza, Allan Felipe Marciano, Alcides Moino Júnior, Daniel de Carvalho Melo Costa, Fernanda da Silva Ferreira, Larah Martins Freitas

**Affiliations:** 1 Federal University of Lavras, Lavras, Brazil; 2 Lallemand Plant Care, Piracicaba, Brazil

**Keywords:** Coffee berry borer, Microbial control, Phagostimulants, Volatiles, IPM

## Abstract

The coffee berry borer (CBB) *Hypothenemus hampei* is the major pest of coffee crops worldwide. The use of prebiotics may help increase the efficiency of *Beauveria bassiana* biological control by enhancing CBB attractiveness or providing a nutritive substrate that may enable the entomopathogenic fungus to initiate the infection process more rapidly. This study evaluated whether the application of *B. bassiana* combined with yeast-fermented prebiotics affects the attractiveness, development, and infestation of CBB in arabica coffee. The first experiment evaluated CBB attractiveness to the prebiotics Preb1 and Preb2 used in traps compared to methanol:ethanol in the field. The second experiment evaluated in the laboratory CBB preference for fruits treated with *B. bassiana* alone and combined with the prebiotics. The third experiment evaluated CBB survival and reproduction in fruits subjected to these treatments in the laboratory. The fourth experiment evaluated treatments application on CBB artificially infested in coffee fruits in the field. As main results, greater numbers of CBB were collected in traps with methanol:ethanol. CBB females preferred fruits treated with *B. bassiana*, *B. bassiana* + Preb1, and *B. bassiana* + Preb2 than control fruits. CBB survival and progeny production were greater in the control than in the other treatments. The prebiotics increased *B. bassiana* efficiency in reducing CBB infestation to coffee fruits only early after application. Although these results suggest potential for the combined use of *B. bassiana* and prebiotics as a CBB management strategy, the effect was limited. New prebiotic formulations and application strategies should be explored to enhance efficacy.

## Introduction

The coffee berry borer (CBB) *Hypothenemus hampei* (Ferrari) (Coleoptera: Curculionidae: Scolytinae) is considered a primary pest of coffee crops worldwide. CBB is present in nearly all coffee-producing countries, causing great damage and yield losses to global coffee production (Johnson et al. 2020). CBB attacks fruits at almost all developmental stages, causing seed weight loss and coffee depreciation (Martinez et al. 2014). The attack may also facilitate the infection of opportunistic microorganisms into coffee seeds, affecting the beverage quality (Vega et al. 2017). The annual losses caused by CBB infestations in the world amount on average US$500 million (Infante 2018).

For the successful management of key pests of agricultural crops such as CBB, integrated pest management (IPM) strategies involving cultural, behavioral, varietal, biological, and chemical control must be adopted along with pest population monitoring (Aristizábal et al. 2002). In Brazil, Mexico, Hawaii, Colombia, and Central American countries, field traps containing the methanol:ethanol kairomone are often used for CBB monitoring because of the high attractiveness of adult females to the volatiles. Based on CBB female sampling using kairomone traps it is possible to estimate the most appropriate timing for application of chemical and microbial insecticides, aiding farmers’ decision-making for CBB control (Aristizábal et al. 2016; Mota et al. 2017; Carvalho et al. 2023; Myrie et al. 2023). The transparent trap baited with methanol:ethanol exhibited the best performance of CBB captures, with an average of 14.3 ± 5.4 adults/trap/week (Carvalho et al. 2023). Although the methanol:ethanol kairomone is efficient in attracting CBB females, few studies have been conducted to assess novel biological attractants for use in CBB monitoring, mass collection, and attract-and-kill strategies.

The most widely used microbial insecticides for pest control are based on the entomopathogenic fungus *Beauveria bassiana* (Bals.) Vuill. (Hypocreales: Cordycipitaceae), which has a broad action spectrum against various insect pests, including CBB (Jaramillo et al. 2015; Hollingsworth et al. 2020; Moran et al. 2021; Bayman et al. 2021). In addition, *B. bassiana* can naturally occur in the agroecosystems, causing epizootics under favorable climatic conditions (Monzón et al. 2008; Sujeetha and Sahayaraj 2014). In Brazil, the main producer and exporter of arabica coffee worldwide, there are currently 59 commercial products and 6 isolates based on *B. bassiana* registered for CBB control (MAPA 2026).

Different isolates of *B. bassiana* have shown significant CBB mortality (Jaramillo et al. 2015; Mascarin and Jaronski 2016; Bayman et al. 2021). However, the efficacy of *B. bassiana* against CBB can be highly variable depending on environmental conditions, fungal characteristics, product formulations, and application strategies. Ferreira et al. (2022) reported control levels ranging from approximately 20 to 75% under different conditions. Considering the complexity of maintaining the viability of entomopathogenic fungi in the environment, the success of *B. bassiana* in controlling CBB depends on several factors, including UV radiation, temperature, humidity, altitude, conidia concentration, and proper application technology (Aristizábal et al. 2016; Vega et al. 2015). Therefore, further studies are needed to evaluate strategies that increase the exposure of CBB colonizing females to *B. bassiana*, as the cryptic behavior of this pest limits fungal contact. Strategies that improve fungal exposure and persistence in the field may enhance the biological control of CBB.

Prebiotics are specific nutrients defined as “selectively fermented ingredients that allow for specific changes both in the composition and activity of microbiota, benefiting host well-being and health” (Gibson et al. 2017). The current prebiotic definition originated from human gut microbiota research and applies well to animal nutrition, but its use for plant, soil, and broader agricultural formulations remains incomplete and still needs sector-specific validation. Prebiotics may potentially provide substrates that support the growth and establishment of entomopathogenic fungi, which could improve pest control efficacy. However, this potential interaction remains to be further investigated.

Some prebiotics are by-products of fermentation by the yeast *Saccharomyces cerevisiae*, owing to a composition rich in amino acids, proteins, and vitamins. Prebiotics may cause insect attraction due to the presence of volatile attractants and phagostimulants in the formulation. However, there is limited information on the use of prebiotics within the context of IPM.

In this study, we evaluated whether the combined application of *B. bassiana* with yeast-fermented prebiotics could enhance the attraction and mortality of colonizing CBB females to treated coffee fruits in laboratory and field conditions. In addition, we evaluated whether the prebiotics could be attractant to CBB females when used in traps on coffee plants in the field.

## Materials and Methods

### Insects used in Experiments

Specimens of CBB used in the experiments were obtained from infested coffee fruits collected in experimental areas with a history of infestation at the Universidade Federal de Lavras (UFLA), Minas Gerais, Brazil. The collected insects were used to establish a laboratory colony that was reared on Cenibroca artificial diet (Giraldo-Jaramillo and Parra 2018) based on green arabica coffee powder. The insects were disinfected with 2.5% sodium hypochlorite for 30 s before introducing to the colony. The colony was kept in a growth chamber under controlled conditions (25 ± 2°C; RH 60 ± 10%; 24 h scotophase) in the Laboratory of Plant Resistance and Integrated Pest Management of the Department of Entomology at UFLA.

### Products used in Experiments

The products used in the experiments consisted of the entomopathogenic fungus *B. bassiana* IBCB 66 (1 × 10^10^ CFU g^−1^, Granada®, Lallemand Biological Solutions Ltda., Piracicaba, MG, Brazil), and two prebiotics in test phase, Preb1 and Preb2, derived from yeast (*Saccharomyces cerevisiae*) submerged fermentation in a predominantly aerobic process. The prebiotics were evaluated as the undiluted crude fermented broth. In the first field experiment, the positive control consisted of the kairomone composed of the methanol:ethanol (1:1) mixture (Dinâmica Química Contemporânea, Ltda.), prepared from high-purity compounds, and added in traps that were hung on coffee plants in the field. The other products were used as treatments in all four experiments, which are detailed in the following sections.

### Experiment 1: Attractiveness of CBB to Prebiotics used in Traps

Field experiments were conducted during December 2021 and January 2022 in areas cultivated with arabica coffee at two locations in the municipality of Lavras, Minas Gerais, Brazil, at an average altitude of 910 m. The fruits were at the green stage, and this period of evaluation was chosen as it is considered the flight period of colonizing CBB females in the region. The first location was in an experimental area at UFLA (21° 22′ 82″ S, 44° 95′ 78″ W), and the second on the commercial farm “Cafua” (21° 10′ 11″ S, 44° 58′ 37″ W). The region has a subtropical climate, with dry winters and hot summers (Dantas et al. 2007), and the soil type is classified as red latosol. The plants in the field were cultivated in 3.3 × 0.6 m spacing between rows and plants, respectively.

The treatments evaluated in this experiment were as follows: control (distilled water), the methanol:ethanol (1:1) kairomone, and the prebiotics Preb1 and Preb2. Treatments were placed in glass vials (10 ml) closed with rubber cap with a hole to allow the emission of volatiles, which were installed in traps. The traps were made with PET bottles (Fernandes et al. 2014) painted with red paint, as this color was demonstrated to be more attractive to CBB (Dufour and Frérot 2008). They were fixed to the orthotropic branch of arabica coffee plants with wire in the middle canopy position, ~ 1.5 m above the ground. The traps were installed with the lids of PET bottles facing down to facilitate collection of insect samples. A 10% detergent solution was added to water in the bottom of traps to disrupt the surface tension and facilitate insect immersion, and 10% sodium chloride was added for aiding insect preservation.

Treatments were arranged in a randomized block design such that four treatments and four replicates were evaluated in both field areas. In the experiment conducted at UFLA, two traps were installed in each replicate, while three traps were installed per replicate on the commercial farm. The average insect collections in each replicate were calculated as a function of the number of traps. The traps representing the same replicate in the block were installed on plants arranged in adjacent planting rows. Traps containing different treatments were spaced 20 m apart, while an 80-m distance was used between replicates.

The number of CBB females captured in the traps was evaluated every 10 days after installation (DAI). In the UFLA area, four evaluations were performed, while on the commercial farm three evaluations. In each evaluation, CBB females captured in the trap solutions were collected and stored in labelled plastic containers containing 70% alcohol. Both the attractants in the glass vials and the solutions at the bottom of traps were replenished in each evaluation.

### Experiment 2: Preference of CBB for Coffee Fruits Treated with *B. bassiana* and Prebiotics

Arabica coffee fruits at the green-cane stage were collected in experimental areas at UFLA and taken to the laboratory, where a free-choice preference assay was performed in a climate-controlled room (25 ± 2°C; RH 60 ± 10%; 12L:12D h). The fruits were collected from plants where insecticide was not applied during that crop season. The bioassay was conducted in Petri dishes (15 cm diameter) lined with dry filter paper.

Four treatments were evaluated for the preference of CBB females: control (water); *B. bassiana*; *B. bassiana* + Preb1; and *B. bassiana* + Preb2. Coffee fruits were immersed for 60 s in the treatment suspensions. For the treatments containing *B. bassiana*, the commercial product Granada® (1 × 10^10^ CFU g^−1^) was used at the manufacturer’s recommended dose (300 g ha^−1^), with the application volume proportional to 400 L ha^−1^. The prebiotics Preb1 and Preb2 were prepared using the recommended dose proportional to 3 L in 400 L water. For *B. bassiana* + Preb1 and *B. bassiana* + Preb2 mixtures, the solutions were prepared individually and then mixed right before the fruits were immersed.

Three fruits of each treatment were placed equidistant from each other and close to the edge of Petri dishes, so that the three fruits from the same treatment were grouped together. Next, 12 females of CBB from the laboratory colony were released in the center of the dishes, for a total of 10 replicates. The number of females boring the fruits was recorded after 30 min and 1, 2, 3, and 24 h. In addition, the number of boring holes and the number of insects inside the fruits were evaluated after 48 h, when the experiment was ended.

### Experiment 3: Mortality and Reproduction of CBB in Coffee Fruits Treated with *B. bassiana* and Prebiotics

The mortality and reproduction of CBB were evaluated after the exposure of colonizing females to treated coffee fruits. The bioassay was carried out using green-cane stage fruits under controlled laboratory conditions (25 ± 2°C; RH 60 ± 10%; 12L:12D h). The same treatments used in the free-choice preference assay were evaluated: control (water), *B. bassiana*, *B. bassiana* + Preb1, and *B. bassiana* + Preb2.

The fruits were treated as in the previous experiment and then placed in Petri dishes (5 cm diameter) lined with dry filter paper. One fruit and one female CBB were transferred to each Petri dish, which was considered a replicate. The dishes were placed on a bench in the laboratory in a complete randomized design, and a total of 50 replicates were prepared per treatment. For evaluations of CBB mortality and reproduction, 25 dishes were randomly chosen for the evaluation at 16 days after application, and the other 25 dishes were evaluated at 25 days. This methodology was used due to the cryptic habit of CBB in developing in the coffee seeds, requiring destructive fruit evaluations for inspection.

On both evaluation dates, the fruits were cut open with the aid of a scalpel, proceeding in the same way for the seeds to allow for proper visualization of the CBB biological forms. The evaluations were conducted under a stereoscope (40 × magnification), recording the number of live adults relative to the total number of adults used in the experiment, as well as the numbers of eggs and larvae in the seeds.

### Experiment 4: Infestation of CBB in Coffee Fruits Treated with *B. bassiana* and Prebiotics in the Field

This field experiment was conducted during March 2022 on the commercial farm “Fonseca” in the municipality of Lavras, Minas Gerais, Brazil, located between the geographical coordinates of 21º 16′ 54.7" S and 44º 55′ 52.5" W. During the experimental period, the mean temperature was 25.2°C (20.2–26.8°C), with mean maximum and minimum temperatures of 30.6°C (25.0–32.6°C) and 19.8°C (15.4–21.4°C), respectively. Mean relative humidity was 41.9% (31.0–49.5%), and cumulative precipitation was 11.0 mm. The soil type is classified as red latosol (Ribeiro et al. 2018). The cultural practices adopted by the coffee farmer followed the recommendations for the state of Minas Gerais (Ribeiro et al. 1999; Silva et al. 2025). The experiment was performed using arabica coffee plants free of insecticide and fungicide applications for at least 30 days, and the fruits were at the green-cane stage. The same four treatments of the laboratory experiments were evaluated: control (water), *B. bassiana*, *B. bassiana* + Preb1, and *B. bassiana* + Preb2. This experiment was performed using CBB artificial infestation to allow for a more homogeneous pest distribution among the plants.

The treatments were arranged in a complete randomized block design, totaling four treatments and six replications. The plants were grown in a 3.0 × 0.8 m spacing between rows and plants, respectively, and each experimental plot was composed of eight plants in a row. A 20-m border was used between plots and the blocks used as replicates were also separated by 20 m from each other. The spraying of treatments was performed using a backpack motorized atomizer with 400 L ha^−1^ water volume.

After spraying of treatments, two plagiotropic branches located at the plant middle canopy position with 30 fruits without signs of CBB infestation were randomly chosen, where voile-fabric cages (30 × 15 cm) were installed confining each branch. Eight plants were used as experimental units in each replicate. CBB females were collected from the laboratory colony and artificially infested at the proportion of 15 females per cage, being the cages closed with a plastic twist tie. The evaluations of CBB infestation in the coffee fruits were performed at 7 and 14 days after application (DAA). In each evaluation date, the cages and enclosed fruits were removed from four plants per plot, and the collected fruits were brought to the laboratory for assessments of the numbers of fruits with CBB live adults, live adults, and bored fruits.

### Statistical Analysis

Data were checked for residual distribution and homogeneity of variances using the Shapiro–Wilk and Levene tests, respectively. For the field experiment with traps, the numbers of CBB females captured were square-root (x + 1)-transformed and analyzed using repeated-measures ANOVA, accounting for the effects of treatments, evaluation time, and treatments x evaluation time. The means of treatments were compared using Fisher's LSD test (α = 0.05).

In the free-choice assay, the numbers of CBB females boring into coffee fruits were recorded at each evaluation time (30 min, 1, 2, 3, and 24 h after release). Data were analyzed using repeated-measures ANOVA considering the effects of treatment, time, and their interaction. The means of treatments were compared using Tukey's test (α = 0.05). The numbers of boring holes and insects inside the fruits were analyzed using ANOVA, and treatment means were compared using Fisher's LSD test (α = 0.05).

In the no-choice assay, the survival data of CBB were analyzed using a generalized linear model (GLM) with Binomial distribution and logit link function. The mean survival rates between treatments were compared using Tukey’s test (α = 0.05). The mean numbers of eggs and larvae were log (x + 1)-transformed and analyzed using ANOVA, with treatment means compared using Tukey's test (α = 0.05). The efficiencies of adult CBB control and offspring reduction were calculated by Abbott’s formula (Abbott 1925).

Data of CBB infestation in coffee fruits after treatment application in field-grown plants were not normally distributed and were analyzed using GLM with Poisson distribution and log-link function. The means of treatments were compared using Tukey’s test (α = 0.05). All data analysis was performed using Statistica v.7 (Statsoft 2004).

## Results

### Experiment 1: Attractiveness of CBB to Prebiotics used in Traps

**Fig. 1:**
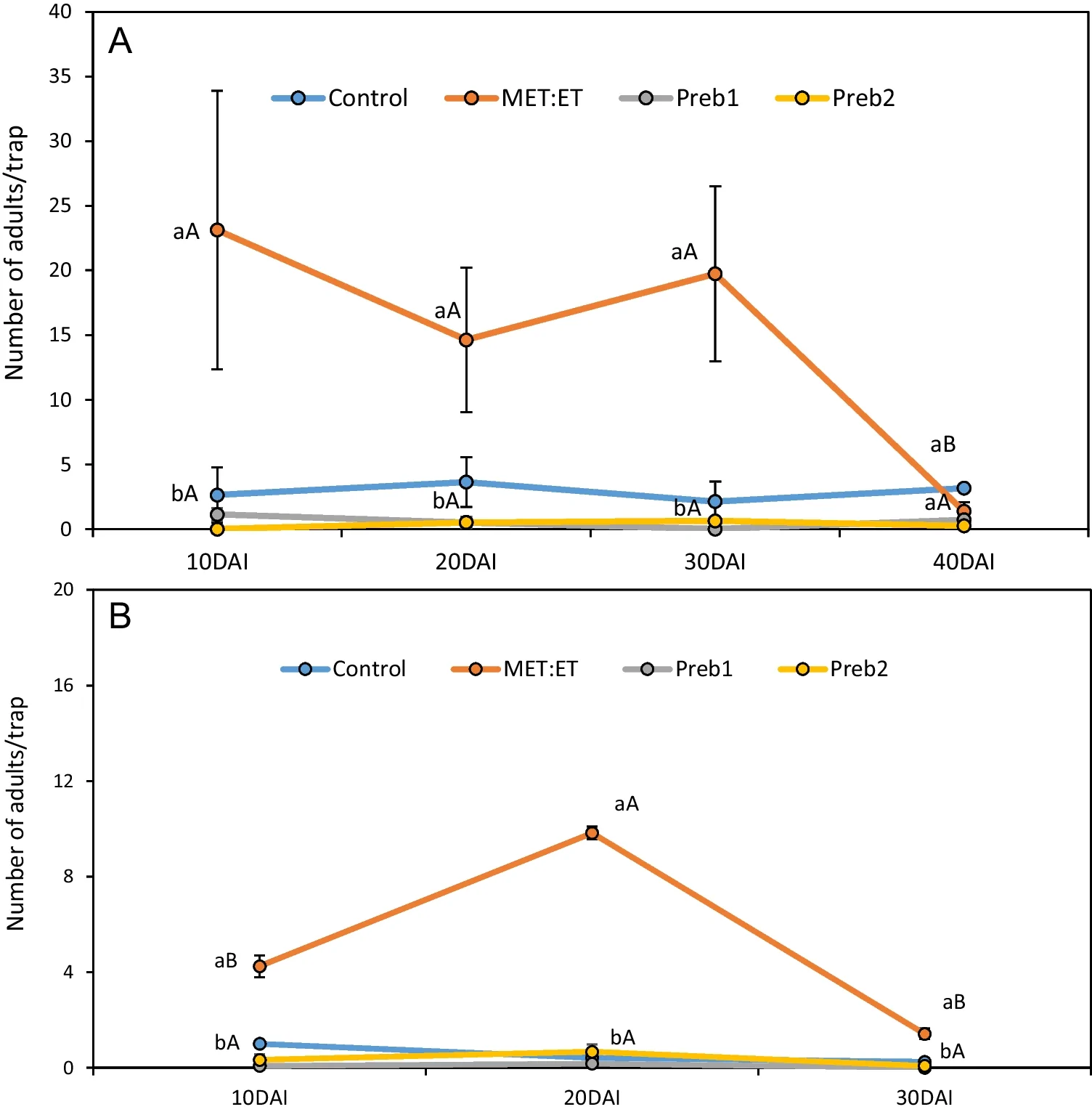
Mean number (± SE) of *Hypothenemus hampei* females collected per trap as a function of treatment and evaluation time at the UFLA experimental site (**A**) and a commercial coffee farm (**B**). Treatments were: Control, MET/ET (methanol:ethanol), Preb1 (Prebiotic 1), and Preb2 (Prebiotic 2). Lowercase letters indicate significant differences among treatments within each evaluation time, whereas uppercase letters indicate significant differences among evaluation times within each treatment (*P* < 0.05)

There was a significant effect of treatment x evaluation time in the number of CBB females captured in both the experimental areas at UFLA (*F*_9, 36_ = 2.84; *P* = 0.0124) (Fig. 1A) and on the commercial farm (*F*_6, 24_ = 7.08; *P* = 0.0002) (Fig. 1B). Greater numbers of CBB were collected with the methanol:ethanol kairomone, differing from the prebiotics and control in all the evaluations in both sites, except at 40 days at UFLA, where there was no difference among treatments.

In the UFLA site, the numbers of CBB females collected at 10, 20, and 30 days after installation (DAI) of traps were similar and significantly greater than at 40 DAI (Fig. 1A). On the commercial farm, the numbers of CBB females collected at 20 DAI was greater than those collected at 10 and 30 DAI, which did not differ (Fig. 1B).

### Experiment 2: Preference of CBB for Coffee Fruits Treated with *B. bassiana* and Prebiotics

**Fig. 2:**
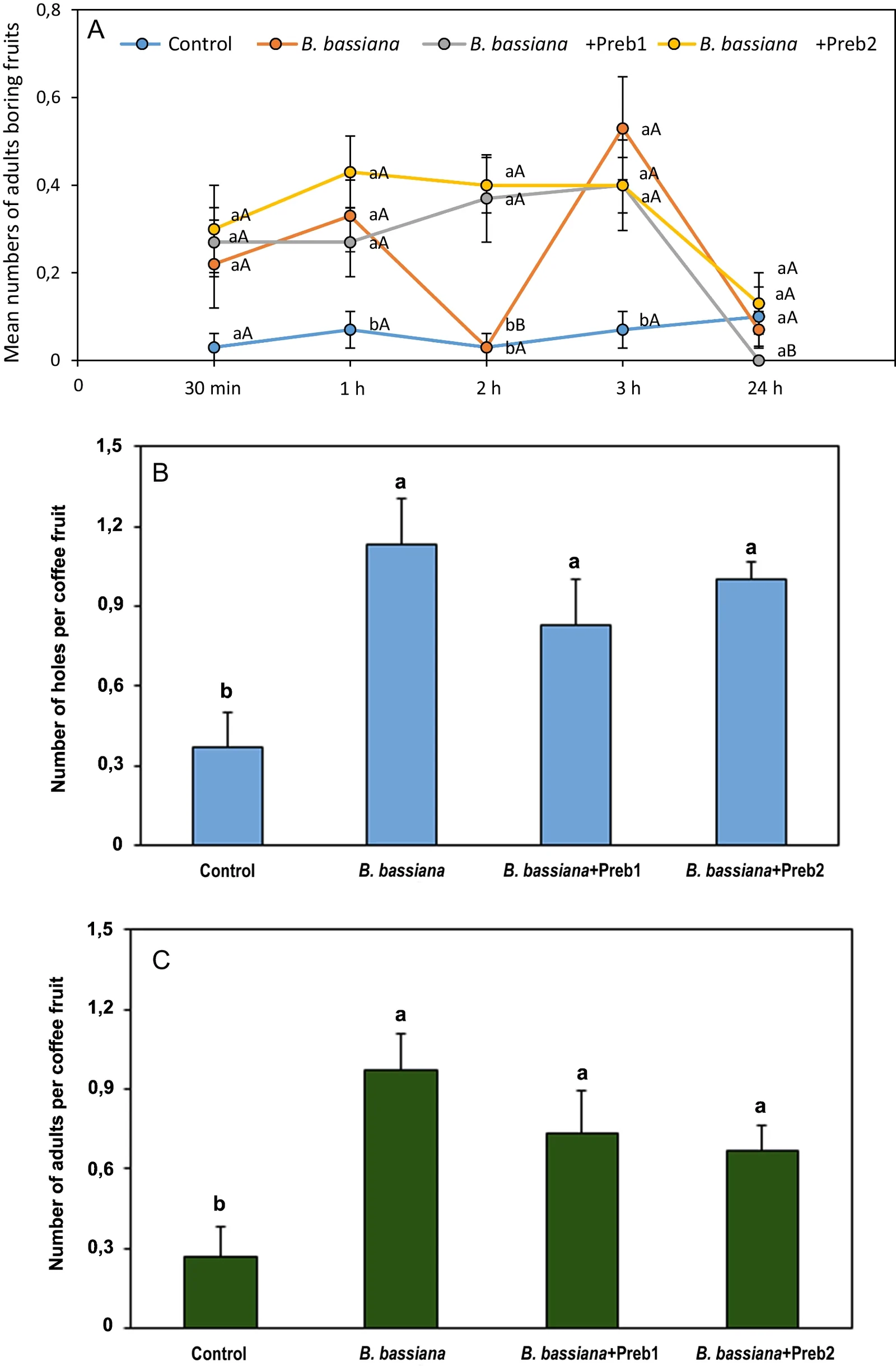
Mean number (± SE) of *Hypothenemus hampei* females boring coffee fruits over time (30 min, 1 h, 2 h, 3 h, and 24 h) in a free-choice assay (**A**), holes per fruit (**B**), and females per fruit (**C**) at 48 h after treatment application. Treatments were: Control, *B. bassiana*, *B. bassiana* + Preb1 and *B. bassiana* + Preb2. Different lowercase letters indicate significant differences among treatments within each evaluation time, whereas different uppercase letters indicate significant differences among evaluation times within each treatment (*P* < 0.05)

The number of CBB females boring into coffee fruits was significantly affected by treatment (*F*_3, 36_ = 6.22; *P* = 0.0016), evaluation time (*F*_4, 144_ = 8.87; *P* < 0.0001), and the treatment × time interaction (*F*_12, 144_ = 2.95; *P* = 0.0011) (Fig. 2A). At 30 min, no differences were observed among treatments. At 1 h, fewer females were boring into control fruits than in those treated with *B. bassiana*, either alone or combined with Preb1 and Preb2. At 2 h, the control and *B. bassiana* alone showed fewer females boring into the fruits than the *B. bassiana*-prebiotic combinations. At 3 h, treatments with *B. bassiana* alone and combined with the prebiotics exhibited more females boring into fruits than the control. At 24 h, there was no significant difference among treatments. Across evaluation times, the number of females boring into coffee fruits remained unchanged in the control and *B. bassiana* + Preb2. In contrast, fewer females were observed at 2 h for *B. bassiana* alone, and at 24 h for *B. bassiana* + Preb1, compared with the other evaluation times for these treatments.

After 48 h, higher numbers of boring holes (*F*_3, 36_ = 4.91; *P* = 0.0058) (Fig. 2B) and CBB adults inside the fruits (*F*_3, 36_ = 4.57; *P* = 0.0082) were observed in the treatments with *B. bassiana*, either alone or combined with the prebiotics, relative to control fruits (Fig. 2C). The two *B. bassiana*-prebiotic combinations did not differ from each other.

### Experiment 3: Mortality and Reproduction of CBB in Coffee Fruits Treated with *B. bassiana* and Prebiotics

**Fig. 3:**
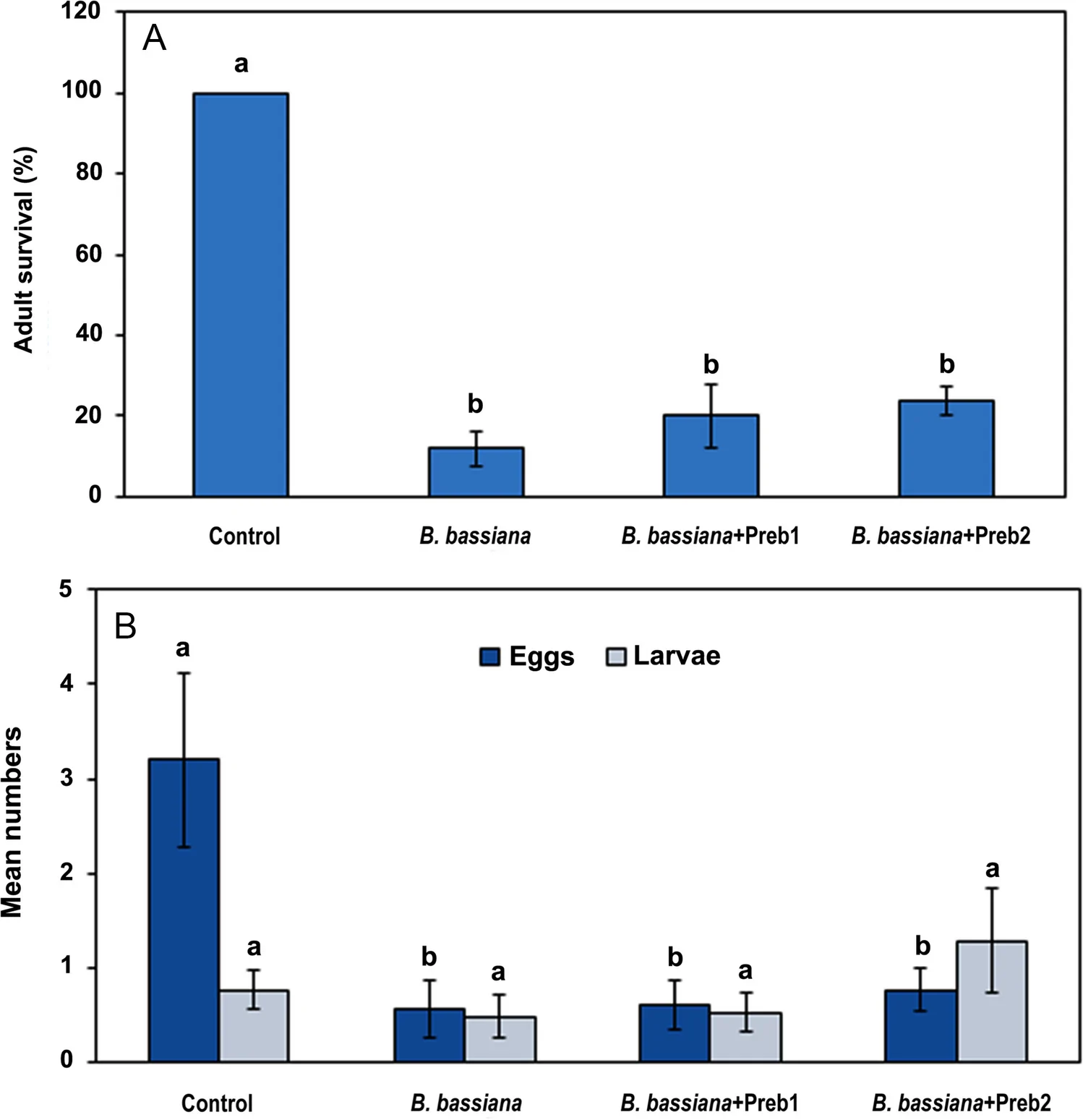
Mean percentage survival (± SE) of *Hypothenemus hampei* females (**A**), and numbers (± SE) of eggs and larvae (**B**) at 16 days after treatments application. Treatments were: Control, *B. bassiana*, *B. bassiana* + Preb1 and *B. bassiana* + Preb2. Different letters denote significant differences (*P* < 0.05)

In the first fruit evaluation at 16 days, the survival of colonizing CBB females was significantly greater in the control than in *B. bassiana*, *B. bassiana* + Preb1, and *B. bassiana* + Preb2 (*X*^2^ = 62.15; *P* < 0.0001) (Fig. 3A). The control efficiency rates of CBB obtained with *B. bassiana*, *B. bassiana* + Preb1, and *B. bassiana* + Preb2 were 88, 80, and 76%, respectively.

**Fig. 4:**
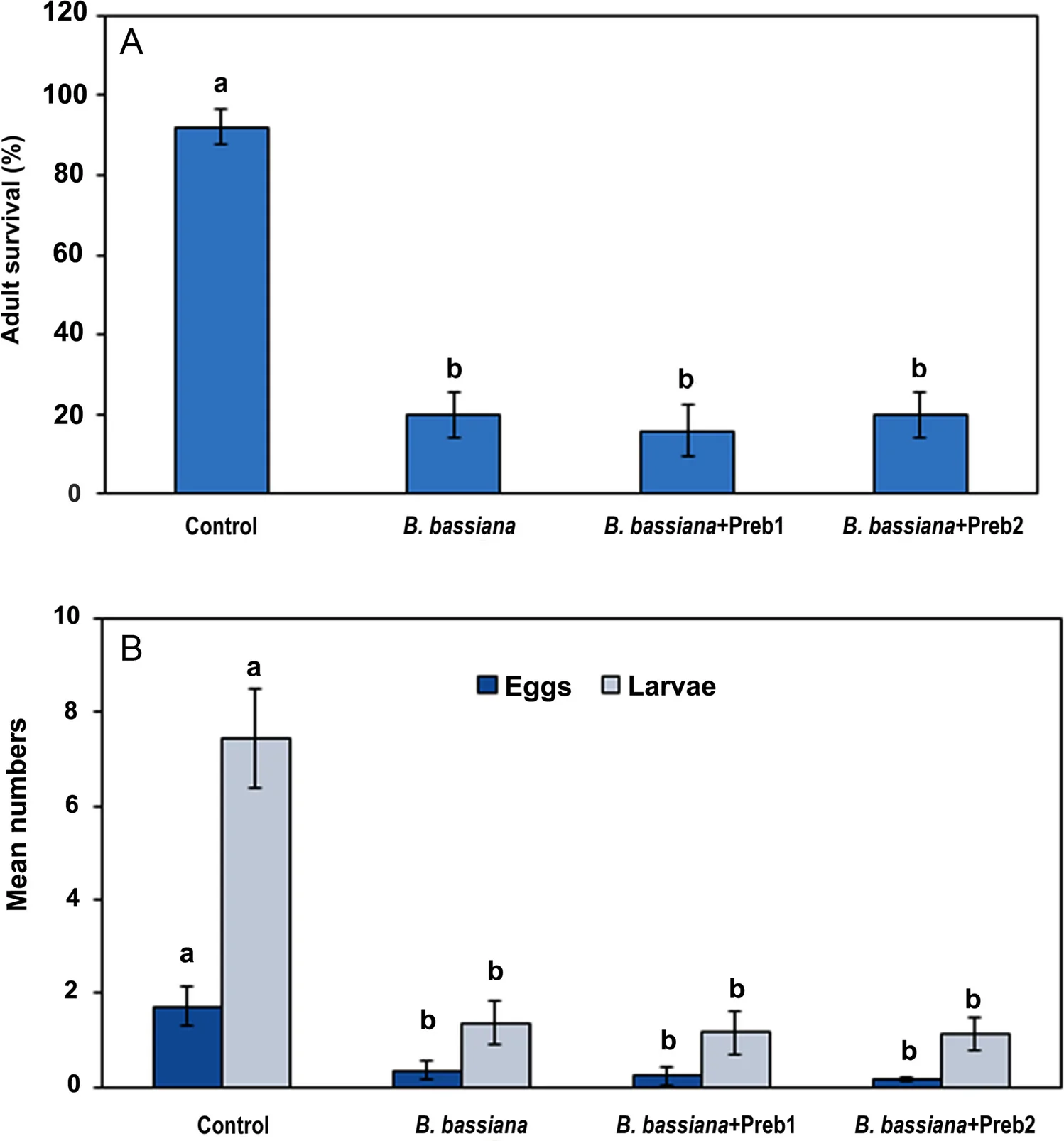
Mean percentage survival (± SE) of *Hypothenemus hampei* females (**A**), and numbers (± SE) of eggs and larvae (**B**) at 25 days after treatments application. Treatments were: Control, *B. bassiana*, *B. bassiana* + Preb1 and *B. bassiana* + Preb2. Different letters denote significant differences (*P* < 0.05)

The number of CBB eggs was significantly higher in the control compared to the treatments with *B. bassiana*, *B. bassiana* + Preb1, and *B. bassiana* + Preb2 (*F*_3, 16_ = 4.23; *P* = 0.0222), which did not differ (Fig. 4B). The efficiencies of oviposition reduction in these treatments were 83, 81, and 76%, respectively. There was no difference in the number of CBB larvae among treatments after 16 days (*F*_3, 16_ = 0.72; *P* = 0.5556) (Fig. 3B).

In the second fruit evaluation after 25 days, the survival data corroborated the first evaluation date and were significantly greater in the control fruits than in the other treatments (*X*^2^ = 24.37; *P* < 0.0001). The control efficiency rates of CBB adults for *B. bassiana*, *B. bassiana* + Preb1, and *B. bassiana* + Preb2 were 78, 83, and 78%, respectively (Fig. 4A).

The numbers of eggs (*X*^2^ = 9.53; *P* = 0.0230) and larvae (*F*_3, 16_ = 10.49; *P* = 0.0005) in the fruits after 25 days significantly differed among treatments. In all treatments there was lower offspring production than in the control. The efficiencies of oviposition reduction with *B. bassiana*, *B. bassiana* + Preb1, and *B. bassiana* + Preb2 were 79, 86, and 91%, respectively. For larval production, the reduction efficiencies were 82, 84, and 85%, respectively. The numbers of CBB eggs and larvae did not differ among treatments containing *B. bassiana* (Fig. 4B).

### Experiment 4: Infestation of CBB in Coffee Fruits Treated with *B. bassiana* and Prebiotics in the Field

**Fig. 5:**
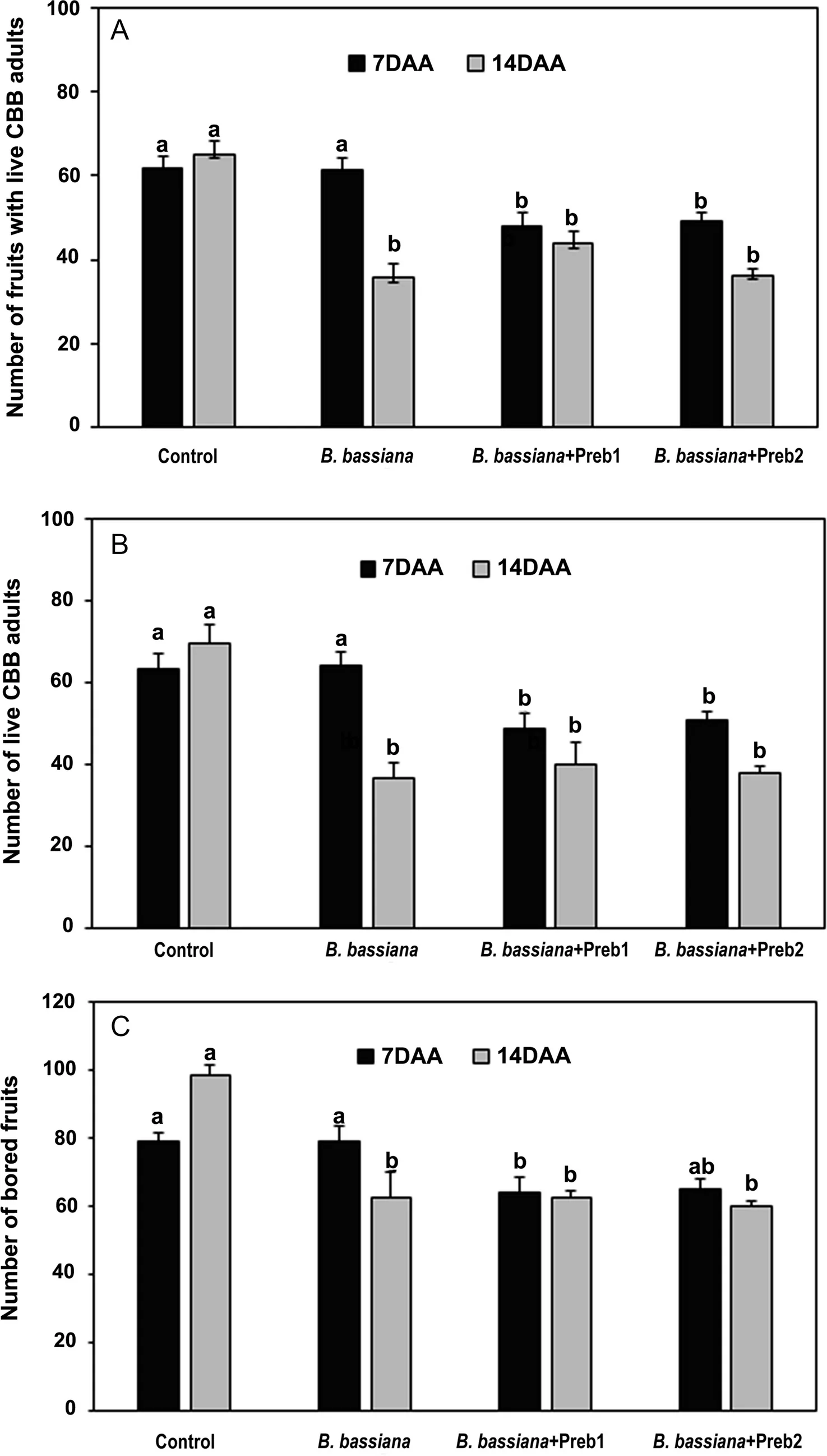
Mean numbers (± SE) of coffee fruits with live *Hypothenemus hampei* adults (**A**), live adults (**B**), and bored fruits (**C**) at 7 and 14 days after treatments application. Treatments were: Control, *B. bassiana*, *B. bassiana* + Preb1 and *B. bassiana* + Preb2. Different letters denote significant differences (*P* < 0.05)

There were significant differences for the mean numbers of fruits with CBB live adults (*X*^2^ = 18.90; *P* = 0.0003) (Fig. 5A), live adults (*X*^2^ = 21.29; *P* = 0.0001) (Fig. 5B), and bored fruits (*X*^2^ = 18.33; *P* = 0.0004) (Fig. 5C) at 7 DAA. The treatments with *B. bassiana* + Preb1 and *B. bassiana* + Preb2 exhibited lower mean numbers relative to the control and *B. bassiana* alone for all parameters, except for the number of bored fruits, in which *B. bassiana* + Preb2 did not differ from the control and the other treatments. At 14 DAA, all treatments with *B. bassiana* showed significantly lower numbers of fruits with live adults (*X*^2^ = 73.75; *P* < 0.0001) (Fig. 5A), live adults (*X*^2^ = 94.06; *P* < 0.0001) (Fig. 5B), and bored fruits (*X*^2^ = 86.55; *P* < 0.0001) (Fig. 5C) relative to the control. For these parameters, the treatments with *B. bassiana* alone were similar to those combined with prebiotics.

## Discussion

The field experiments with traps demonstrated that in both sites greater numbers of CBB females were collected with methanol:ethanol, differing from the treatments with water (control) and the prebiotics Preb1 and Preb2. The results obtained with methanol:ethanol were expected because this kairomone is often used for monitoring CBB population densities, supporting the decision-making for the pest control in coffee crops in many countries (Aristizábal et al. 2016). On the other hand, the prebiotics herein evaluated did not prove to be attractive to CBB colonizing females in traps under field conditions at any evaluation date or location.

The attractive effect of methanol:ethanol to CBB found in our study was similar to that found by Ortiz et al. (2004). The authors stated that the composition of volatile compounds emitted by coffee fruits is dominated by very high alcohol levels, thus explaining why traps with mixed alcohol solutions are effective for CBB attraction. Similar results were also found by Moreno Rodríguez et al. (2010), where the methanol:ethanol (3:1) mixture was the most effective treatment in capturing CBB females, unlike ethyl alcohol used alone or mixed with roasted ground coffee, which captured fewer females. In the study of Fernandes et al. (2011) with traps containing ethanol, methanol, and benzaldehyde, the authors concluded that their use for CBB monitoring is a viable strategy that allows for easy and low-cost decision-making for the pest control. Fernandes et al. (2014) assessed the use of 30 red PET-bottle traps containing methanol:ethanol per hectare and concluded they were partially effective in CBB mass collection, reducing the percentage of bored fruits by 57%.

Although the Preb1 and Preb2 prebiotics did not attract CBB adults under field conditions, according to the literature no studies have evaluated prebiotic formulations for attracting insects in the context of Integrated Pest Management (IPM). This IPM strategy combining both behavioral and biological control may be promising for CBB when more attractive compounds derived from microbial fermentation are to be used. Thus, new studies that evaluate other prebiotics are needed to better understand the interactions between CBB and these compounds for use as IPM strategies in coffee crops.

The second experiment of this study using a free-choice assay in the laboratory revealed the preference of CBB females for treatments with *B. bassiana*, either alone or combined with the prebiotics, relative to the control. The treatments with *B. bassiana* exhibited greater numbers of fruits with boring holes and adults inside the fruits after 48 h. Although CBB females changed their choice for fruits treated with *B. bassiana* alone during the first hours of evaluation, there was a consistent preference for the fruits treated with the entomopathogenic fungus either alone or combined with the prebiotics in most evaluation times. This greater preference of CBB may be explained by some hypotheses considering the presence of phagostimulants, volatiles, and visual stimuli.

The first hypothesis concerns the presence of additive phagostimulant compounds in the bioinsecticide formulation of *B. bassiana* used (Granada®) that could have made the product more attractive to CBB (Acuña Jiménez et al. 2015). This may explain the differences between the effects of treatments containing *B. bassiana* as compared to control fruits. We are not aware about the presence of any phagostimulant in the commercial bioinsecticide, and the label of the product does not provide information among the inerts, which make up 80% of the formulation composition.

As a second hypothesis, the preference of CBB females for fruits treated with *B. bassiana* may be associated with the release of volatile compounds within the confined microenvironment of the Petri dishes. Although the chemical characterization of the volatiles emitted by the treatments were not performed in the present study, these compounds may have acted as short-range olfactory cues detected by CBB females. For instance, the attraction of *Drosophila melanogaster* to *S. cerevisiae* was shown to be independent of the yeast's growth stage. Attraction remained high and similar during both the respiro-fermentative exponential growth phase and the early and late respiratory phases, even when ethanol concentrations and growth declined (Becher et al. 2018). Emission of the complex blend of *S. cerevisiae* fermentation volatiles, particularly acetate esters, act as important infochemicals in insect attraction (Đurović et al. 2021).

Considering the olfactory and visual stimuli involved in insect-plant interactions, volatile compounds are chemical stimuli easily detected by CBB. The discrimination of colors combined with olfactory stimuli is essential for CBB females when choosing coffee fruits (Mathieu et al. 2001), and it is known that the fruits at all maturation stages emit volatiles (Blassioli-Moraes et al. 2019; Michereff et al. 2018; Rosa‐Cancino et al. 2023). In the study of Mendesil et al. (2009), CBB females were attracted to volatiles from ripe and dried coffee fruits, while volatiles collected from unripe fruits were not attractive, such that CBB did not respond positively to the odor*.* In addition, González-Mas et al. (2021) while evaluating the effect of endophytic colonization of *B. bassiana* in melon and cotton plants confirmed that the fungus-colonized plants emitted a distinct blend of volatiles compared to uncolonized plants, and that some of the identified compounds were reported to cause attraction to natural enemies. Further laboratory and field studies should be conducted to determine whether the application of *B. bassiana* alone and combined with prebiotics to coffee fruits could release distinct volatiles that are attractive or arrestant to CBB at short distances, since the prebiotics used in traps in the field were not attractive at long distances.

The third hypothesis regarding CBB preference is that the whitish color of fruits treated with *B. bassiana* combined or not with prebiotics may have caused visual attraction when detected by the females. When the fruits were immersed in the treatments, they became slightly whitish because the suspensions generated foam and probably changed light reflectance. In addition, when CBB females tasted the treated fruits, phagostimulant compounds in the treatments with *B. bassiana* may have stimulated them to remain on the fruits. According to Vega et al. (2014), the eyes of CBB females evolved more than those of the males because of the need to leave the coffee fruits in search for new ones for colonization. Thus, changes in the light spectrum resulting from the deposition of suspensions containing *B. bassiana* on the coffee fruits may have affected CBB behavior.

The third experiment evaluated the survival and reproduction of CBB females in fruits treated with *B. bassiana*, *B. bassiana* + Preb1, and *B. bassiana* + Preb2 in the laboratory, revealing higher survival in the control, while in the other treatments the rates were low (12 to 24%). These treatments also impaired CBB reproduction, such that lower numbers of eggs and larvae were found after 16 and 25 days, except at the first evaluation that larvae numbers did not differ among treatments because there was not enough time for the eclosion. Although fungal sporulation characteristic of *B. bassiana* was visually noted on dead insects, fungal infection was not systematically recorded and confirmed. Therefore, not all observed mortality can be unequivocally attributed to *B. bassiana*, although the natural mortality in the control was relatively low. The combination of *B. bassiana* with the prebiotics did not increase the control efficiency of CBB females nor reduced the production of eggs and larvae.

Although the nature of the used additives differs, these results are similar to those obtained by Souza et al. (2020), where the emulsifying agents arabic gum and X1 mixed with *B. bassiana* did not increase CBB mortality. Rosas-García (2008) found that the use of phagostimulants is being improved to prevent the insects from ingesting sublethal doses of the fungus and causing low mortality. To the best of our knowledge, there is no information in the literature on the use of prebiotics to increase the efficiency of infection and mortality of insect pests by entomopathogenic fungus. Advances in research using effective additives to increase insect feeding and contact with fungal bioinsecticides are very important for their successful application as an IPM strategy.

The application of *B. bassiana* in our laboratory experiment effectively controlled CBB females and caused sublethal effects on their reproduction, regardless of the presence of prebiotics. The mortality rates found herein are similar to those found by Jaramillo et al. (2015), in which although CBB infected with *B. bassiana* could penetrate the fruits and lay eggs before dying, the generated offspring were lower than those in the control fruits. In another study conducted in Colombia by Vera et al. (2011), the combination of *B. bassiana* isolates decreased CBB population that emerged from coffee fruits fallen on the ground, reducing the size of future generations. Our results were similar to those found in the study of Greco et al. (2018) performed in Hawaii, where the percentage of dead CBB females ranged between 69 and 95%.

The results obtained in the fourth experiment using CBB artificial infestation in the field evidenced the efficacy of *B. bassiana* application alone or combined with the prebiotics Preb1 and Preb2. At the first evaluation 7 days after spraying, the combined application of *B. bassiana* with both prebiotics reduced the numbers of fruits with live CBB adults, live adults, and bored fruits relative to *B. bassiana* alone and control. The exception was for the number of bored fruits with Preb2 that did not differ from the control and the other treatments. At 14 days after spraying, the treatments with prebiotics did not differ from *B. bassiana* single application, but all of them differed from the control for all parameters.

The lower infestation in the treatments containing *B. bassiana* combined with prebiotics at 7 days after application indicate a transient and limited effect of the combinations, since this difference was no longer observed after 14 days. Therefore, the results do not provide consistent evidence that prebiotics enhanced *B. bassiana* efficacy. It is important to mention that this experiment used CBB artificial infestation, and the effects on natural pest colonization could vary. In addition, the mechanisms underlying this response were not investigated in the present study. Therefore, it is not possible to determine whether the observed effect was associated with changes in fungal viability, conidial germination, persistence on fruit surface, insect behavior, or other factors. Further studies evaluating these mechanisms are needed.

According to Kaiser et al. (2019), it is possible to enhance the persistence of *B. bassiana* spores under UV radiation by using formulations with natural additives. The optimization of the prebiotic formulations with these compounds may be critical for improving the efficacy of fungi-based insecticides for the control of CBB and other pests in IPM systems (Vega et al. 2012). Moreover, the maintenance of the efficacy of microbial insecticides over time is fundamental to achieving greater pest mortality, implying in lower application frequency, and higher economic viability.

Information obtained in our study demonstrates that application of *B. bassiana* can provide satisfactory CBB control, representing a sustainable control tactic in IPM systems that should be preferred over the use of toxic chemical insecticides, such as the organophosphate chlorpyrifos. This insecticide accounts for approximately one third of the commercial products registered for CBB in Brazil (MAPA 2026), and it is also allowed in other countries, while others have banned its use in coffee production.

In summary, the yeast-fermented prebiotics Preb1 and Preb2 did not provide attractiveness of CBB in field traps. CBB females preferred coffee fruits treated with *B. bassiana*, either alone or combined with the prebiotics in the laboratory, suggesting possible effects of phagostimulants, volatile compounds, or visual stimuli on CBB behavior. Mixture with the prebiotics did not enhance *B. bassiana* effectiveness in reducing CBB mortality and offspring in the laboratory. The combined application of *B. bassiana* with prebiotics provided an additive effect in reducing CBB infestation in coffee fruits early after field application, but the effect was very limited. Further studies are needed to evaluate new formulations of prebiotics combined with *B. bassiana* and other entomopathogenic fungi for use as IPM strategies against CBB in sustainable coffee crops.

## Data Availability

The data that support the findings of this study are openly available in Mendeley Data at https://data.mendeley.com/datasets/sj6mvndk7m/1, reference number https://doi.org/10.17632/sj6mvndk7m.1.
